# Physiologic Midtreatment Tooth Movement as a Correction Strategy for Iatrogenic Root Exposure

**DOI:** 10.1155/2020/8841009

**Published:** 2020-09-05

**Authors:** Adith Venugopal, Paolo Manzano, M. Srirengalakshmi, Anand Marya, Nikhilesh R. Vaid, S. Jay Bowman

**Affiliations:** ^1^Department of Orthodontics, University of Puthisastra, Phnom Penh, Cambodia; ^2^10:10 Dental Clinic, Manila, Philippines; ^3^Department of Orthodontics, Saveetha Dental College, Tamil Nadu, India; ^4^European University College, DHCC, Dubai, UAE; ^5^Department of Orthodontics, University of Michigan, USA

## Abstract

In the case report showcased, we describe orthodontic treatment of a female patient with an excessive gingival display on smiling and severe dental crowding, with maxillary canines positioned excessively buccal (ectopic) to and in near transposition to the lateral incisors. The treatment involved extractions and initial use of unmitigated forces leading to excessive gingival thinning and buccal root positioning of the ectopic canines. Eventually, the root position was corrected and periodontal equilibrium was attained. On finishing the treatment, all objectives were achieved with a good esthetic outcome as well as excellent dental and occlusal relationships.

## 1. Introduction

Ectopic and buccally positioned canines are frequent occurrences in an orthodontic practice [1]. Various etiologies have been attributed to these ectopic and buccally erupting permanent canines. The most common of them is the lack of sufficient arch perimeter [2, 3], while others attribute this to the long and tortuous path of eruption that is usually buccal to the dental arch. Since they erupt after the laterals and first bicuspids, approximation of their roots may make it harder for the canines to erupt, in turn causing an ectopic eruption, most commonly manifested as an exaggeration in their buccal position [4, 5]. While treating ectopic canines, a biomechanical strategy is of extreme importance. Emphasis should be placed on avoiding unmitigated force systems to prevent iatrogenic complications.

In 1996, Behrents [6] defined iatrogenics as something unintentionally induced by treatment. Incorrect choice of dental procedures, poor treatment indications, adopting unaccounted treatment strategies, under estimation of treatment time, not changing a treatment plan when necessary, overambitious use of temporary anchorage devices as an anchorage strategy [7], and not establishing good communication with the patient are failures that may significantly affect outcomes, quality, and stability of correction. All these factors delay treatment efficiency [8].

Alveolar defects such as a fenestration or dehiscence are some common iatrogenic effects that may result as a consequence of improper force application to the dentition. Alveolar dehiscence is defined as a lack of the facial or lingual alveolar cortical plate, resulting in a denuded root surface [9]. These alveolar defects arise from different predisposing factors such as decreased thickness of the alveolar bone, labial placement of the tooth in the dental arch, contour of the roots, abnormal occlusal factors, orthodontic tooth movement, or periodontal and endodontic pathology. And more often than not, these conditions are commonly associated with the anterior region of the dental arch [10].

## 2. Case History and Diagnosis

**Figure 1:**
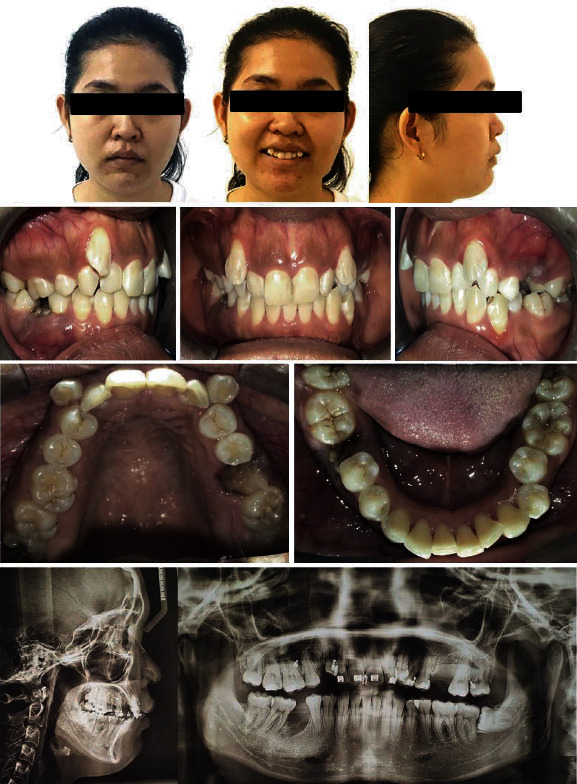
Pretreatment records with radiographs.

A 28-year-old female presented to the office, with the chief complaint of misaligned teeth. Her medical history reported her to be healthy. The clinical examination revealed a convex facial profile with a slightly increased lower third of the face, acute nasolabial angle, slight protrusion and incompetence of the lips, retruded and extruded maxillary incisors, high ectopic canines with a distal crown inclination, excessive display of the gingiva on smiling, and a lot of strain on the lower lip and chin soft tissue area (Figure 1). There was no deviation on mouth opening or mandibular closure. Also, no symptoms of any temporomandibular dysfunctions were reported.

Furthermore, the maxillary canines presented excessively buccal and the roots almost transposed over the laterals. The canines presented with mesial rotations and distal crown inclinations. The mandibular arch presented with moderate crowding and a curve of Spee measuring 3 mm. When in occlusion, the maxillary left lateral incisor showed a dental crossbite, in addition to an overbite of 2 mm and an overjet of 1 mm. The upper midline was coincident with the facial midline, but the lower midline was deviated to the left by 2 mm. The gingival contours of the maxillary anterior segment showed severe lack of leveling. The left mandibular canine and first premolar were almost in a crossbite (Figure 1).

The upper left first molar was freshly extracted because of a severe carious lesion on it. The lower right first molar was mutilated due to a previous carious infection, and the root stumps were referred for extractions prior to placing the brackets. Carious lesions on teeth #36, #37, #47, and #48 were referred for restorations.

Dental radiographic examination revealed the presence of all permanent teeth except #46 and #26. The lower left third molar was impacted and mesially angulated. No pathologic lesions were detected (Figure 1).

The cephalometric analysis (Table 1) showed a forward relationship of the maxilla to the mandible (ANB 8°), with retrusion of the maxilla (SNA 78°) and mandible (SNB 70°), and an increased vertical relationship (SN-MP 41°; FMA 34°). The maxillary incisors were projected palatally (U1-NF 98°), and the mandibular incisors were slightly proclined (IMPA 97°).

## 3. Treatment Objectives

The treatment objectives were to (1) improve the profile of the patient, (2) alleviate the crowding on the maxillary arch and make space for the traction of the ectopically placed canine, (3) establish class I canine relationship on both sides, (4) correct the left lateral incisor crossbite, (5) maintain ideal overjet and overbite, (6) eliminate excessive gingival display on smiling, (7) obtain good interdigitation, and (8) ensure stable results.

## 4. Treatment Alternatives

Due to the presence of localized crowding of the upper anteriors, the presence of maxillary canines with no space to align into the arch, a slightly convex facial profile with protruded upper lips, and an extraction space i.r.t. the already extracted upper left first molar, we proposed orthodontic treatment with extraction of either upper right first or second premolar and use of fixed appliances, followed by retraction and subsequent traction of the upper canines into occlusion with adequate anchorage control using TADs.

If the dental arches were to be leveled and aligned with a nonextraction approach or distalization of posterior teeth, the treatment may have resulted in further lip and bidental protrusions. Any approach involving distalization of teeth in the first quadrant to make space by different approaches (e.g., skeletal anchorage) might prove challenging and time-consuming and would inevitably require the extraction of the third molar.

Considering the patient's protruded lips, convex profile, severe crowding on the upper arch, and lack of space for the ectopic maxillary canines to align, a nonextraction approach may have been unpredictable and lead to further complications.

After carefully considering all the options, it was decided to extract the upper right first premolar instead of the second as it was closer to the ectopic canine and would warrant quicker correction.

## 5. Treatment Progress

This case was treated using 0.022^″^ × 0.028^″^ slot preadjusted edgewise appliances with MBT prescription. Extraction of the upper right first premolar was performed, and interradicular TADs were placed, two of which were placed between the upper left first and second molars (#26-#27) and between the upper right first molar and second premolar (#16-#15).

A segment of the 0.016^″^ NiTi wire was used from #17-#15 and #24-#25 to partially level the upper right and left posterior segments.

A light traction using a power chain measured using a Dontrix gauge to deliver 50 grams of force was applied to the bracket on the tooth #13 from the TAD on the right maxillary segment. A similar force was applied from the TAD on the left to #24 in order to distalize it in order to gain space for ectopic #23.

**Figure 2:**
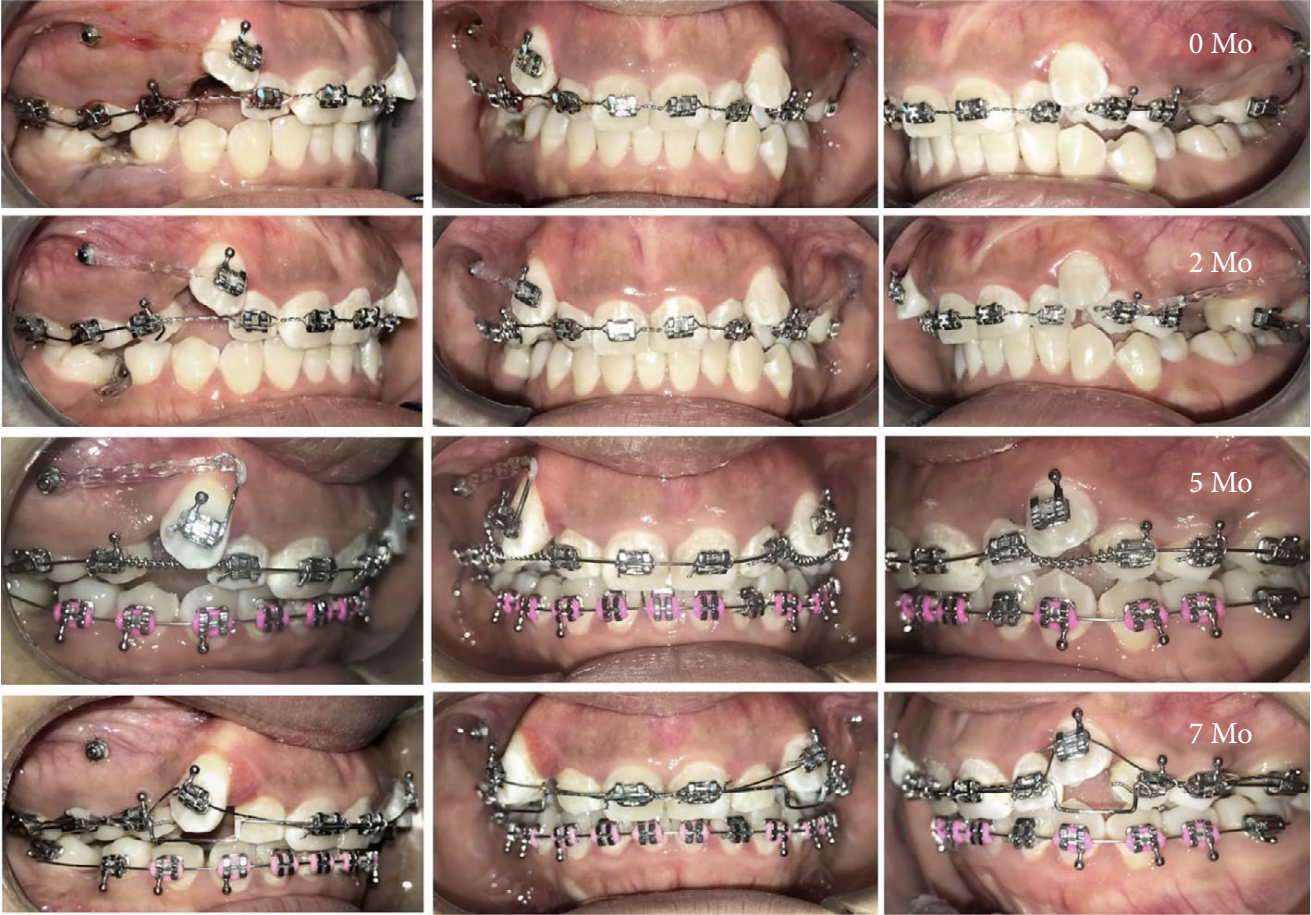
Usage of unmitigated forces causing distal crown tipping and buccalization of #13 root.

On subsequent review, the upper anterior teeth and the molars were included in the treatment using a light 0.014^″^ NiTi wire. It was noticed that the crown of #13 was tipping further distally, and so, it was decided to fabricate an attachment similar to a power arm, on #13 as shown in Figure 2, to approximate the forces closer to the CR of #13 in order to facilitate more bodily movement. To create more space for the left upper canine and to maintain space for the right canine, an open nickel titanium spring was compressed between #15-#12 and #22-#24.

Although efforts were made to reach closer to the CR of #13, further distal tipping along with increased root prominence was noticed. Once adequate space was achieved on both maxillary segments to get the canines into occlusion, the space was maintained using a rectangular stainless steel 0.016^″^ × 0.022^″^ bypass archwire and an overlay 0.012^″^ NiTi wire was used for the further traction of canines into the arch (Figure 2).

**Figure 3:**
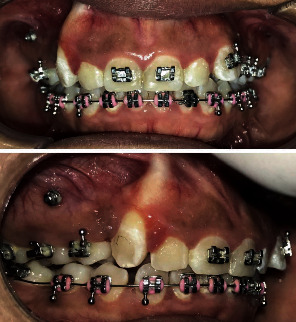
Extreme gingival thinning over the root of #13.

**Figure 4:**
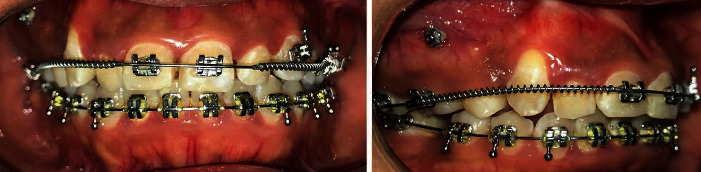
Three months of active space opening and spontaneously letting the canine root move into the alveolar bone.

After 7 months of active treatment, excessive gingival thinning over the root of #13 clearly indicated active dehiscence of the alveolar bone (Figure 3). It was then decided to remove all active forces from tooth #13. Brackets were removed from teeth #12, #13, #15, and #22, and nickel titanium open coil springs were placed between #16-#11 and #21-#24 to gain spaces and relieve the pressure on #13. Stops were placed distal to #11 and #21 in order to prevent excessive proclination as a side effect of the active open coil springs. Follow-up reviews over the next three months showed reduced root prominence over #13 indicating that the root moved physiologically into the alveolar housing (Figure 4).

**Figure 5:**
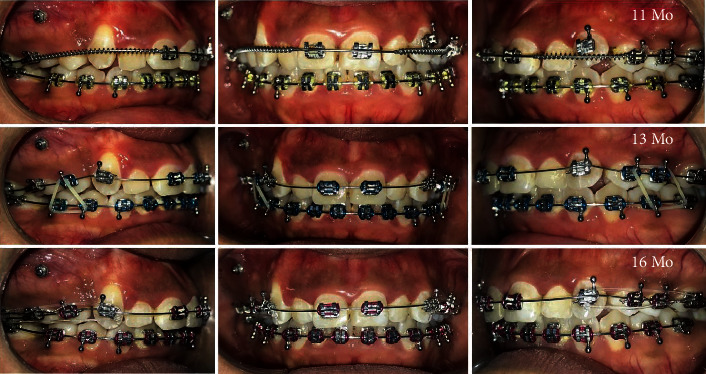
Light forces being used after spontaneous relapse of #13 into the bone in order to achieve intercuspation.

**Figure 6:**
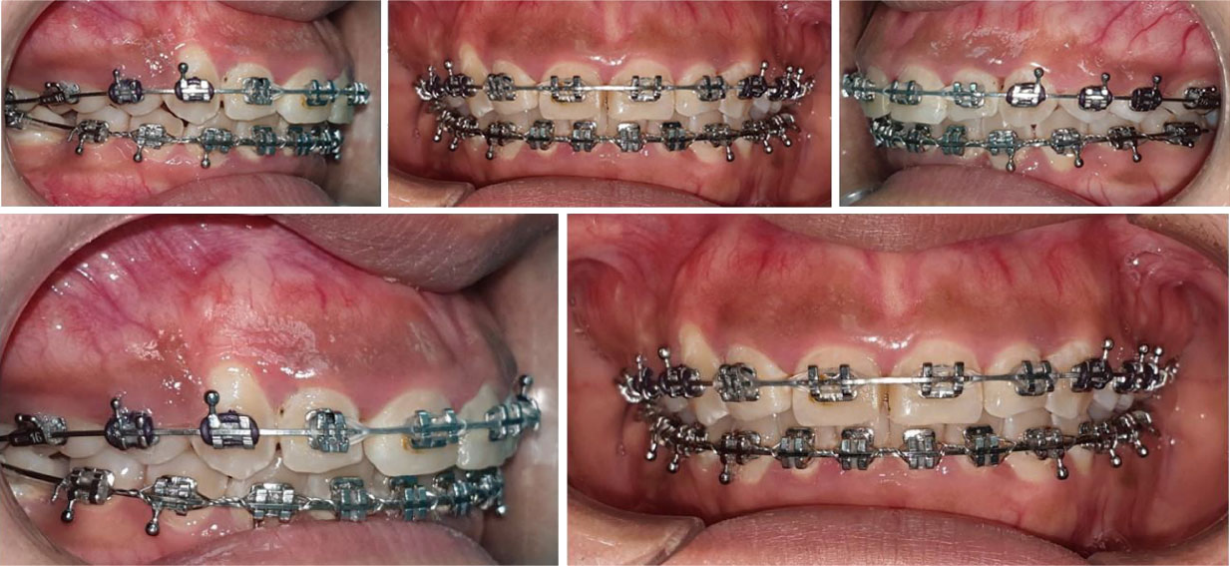
Finishing with coordinated 0.019^″^ × 0.025^″^ stainless steel archwires.

Brackets were now rebonded on #15 and #13, and a flexible continuous 0.014^″^ NiTi archwire was placed on the upper arch with light intermaxillary elastics for occlusal settling (Figure 5). Once #13 was brought vertically into occlusion, a stiff 0.021^″^ × 0.025^″^ NiTi archwire was inserted to correct all the third-order discrepancies. (Figure 6).

Symmetric and coordinated stiff archwires (0.019^″^ × 0.025^″^ stainless steel) were used for finishing stages, maintaining the original dental arch form. This phase required enhancing buccal root torque on the maxillary lateral incisors (Figure 6). Removable wraparound retainers were used on the upper and lower dentitions alongside fixed lingual bonded retainers to retain the orthodontic corrections.

**Figure 7:**
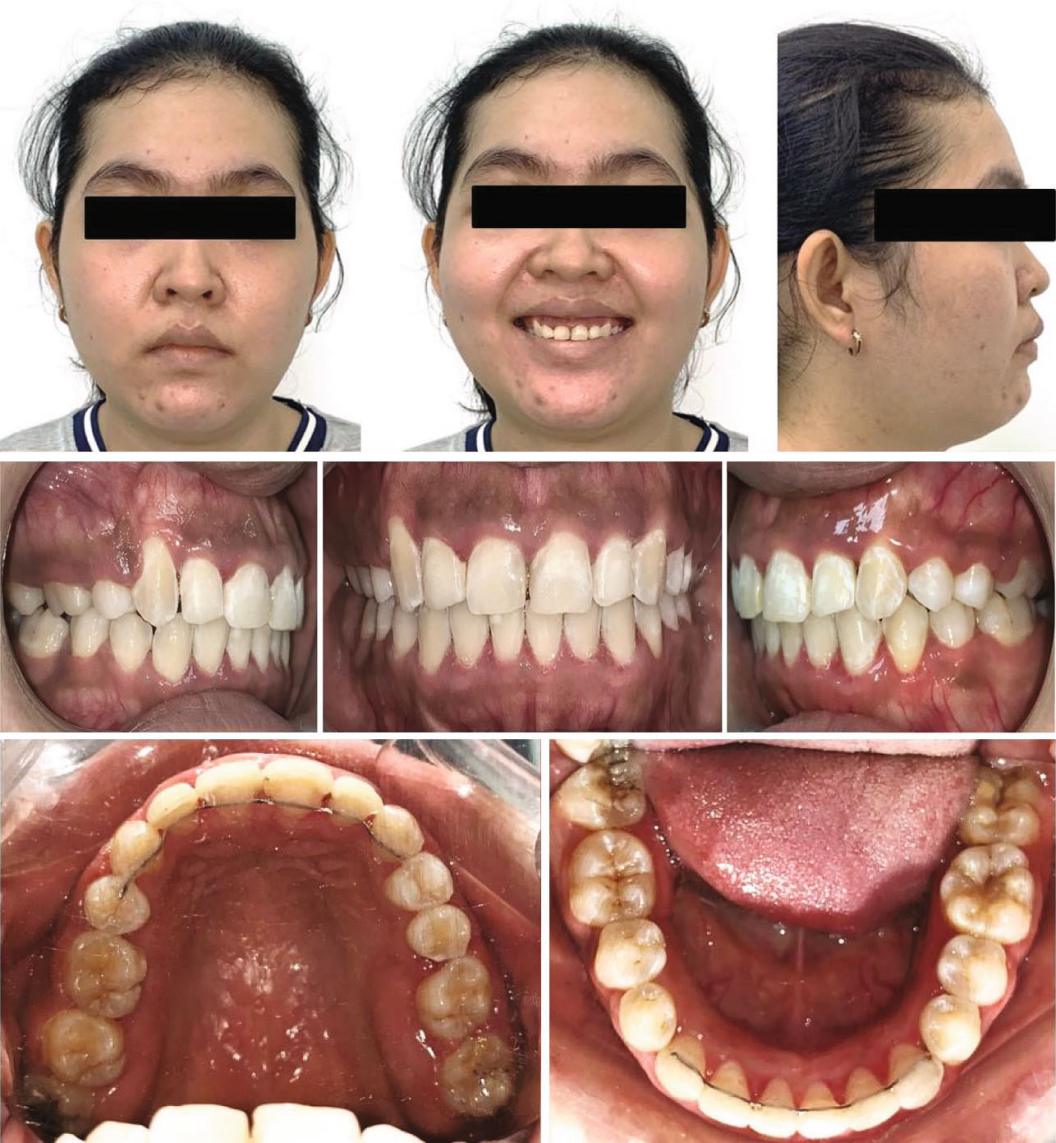
Posttreatment extraoral and intraoral pictures prior to gingivectomy.

**Figure 8:**
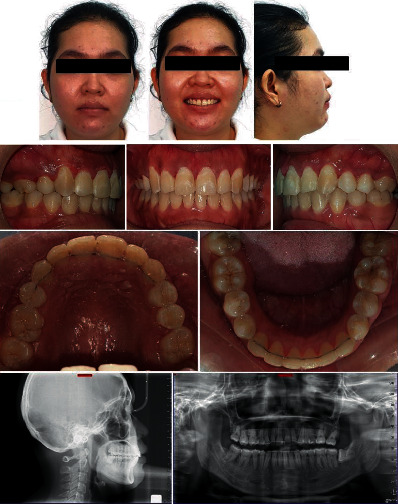
Posttreatment extraoral and intraoral pictures with radiographs after gingivectomy.

Gingivectomy was performed on the upper dentition to even the gingival contours on all teeth and at the same time reduce the gingival display on smiling (Figures 7 and 8).

## 6. Treatment Results

Allowing physiologic midtreatment tooth movement of the upper right canine into the alveolar bone as a correction strategy for the iatrogenic root exposure enabled excellent gingival and periodontal harmony around the canine with stable results (Figures 7 and 8). There was no blanching or partial see through of the root contour through the gingiva over #13 at the end of treatment. Also, there was no tenderness or root prominence felt over #13 on palpation.

Satisfactory outcomes were achieved with an improved facial profile and smile harmony (Figure 8). Optimal occlusal contacts were obtained between all of the other teeth, especially the canines. Overjet and overbite were maintained, and the midlines were now coincident with each other and with the facial midline (Figures 7 and 8). A balanced gingival contour along with optimal gingival display on smiling was obtained, particularly by leveling the gingival margin of the upper dentition by performing a gingivectomy in the end (Figure 8).

**Figure 9:**
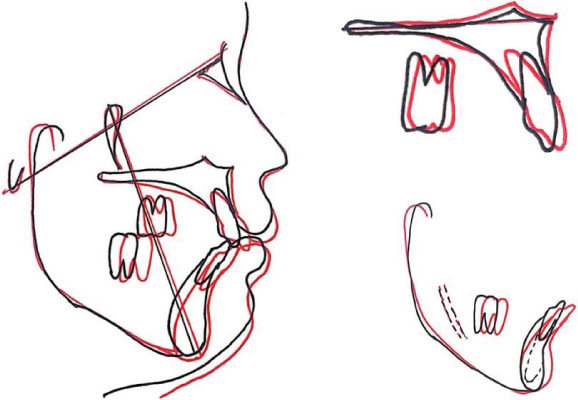
Superimpositions of pre- and posttreatment cephalograms.

Radiographic analysis (Figures 8 and 9; Table 1) revealed a good appearance of the alveolar ridges and bone, as well as appropriate root parallelism. The cephalometric analysis (Figure 8; Table 1) and superimposition of the tracings (Figure 9) indicate a forward relationship of the maxilla to the mandible with minimal changes in the chin position posttreatment (ANB 7, SNA 79, and SNB 72).

**Figure 10:**
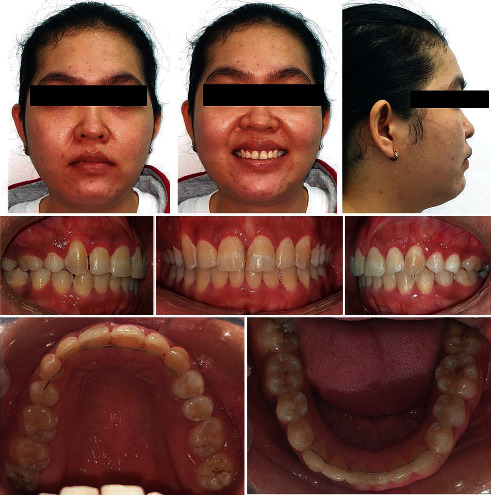
Treatment records at one-year follow-up review.

Improvement was noted in the facial profile and position of the lips with reduced mentalis muscle strain due to reduction in the lower facial height (pre-ANS-GN 68; post-ANS-GN 66). Superimposition showed a slight counterclockwise rotation of the mandible (pre-FMA 34; post-FMA 33). Upper incisors showed a good axial inclination compared to the pretreatment position (pre-U1-NF 98; post-U1-NF 105). Lower incisors showed a 2° proclination posttreatment. Furthermore, there was minimal loss of anchorage in the upper and substantial amount in the lower molars (Figure 9). Excellent gingival and periodontal harmony around the canine with stable occlusal contacts was documented even one year after removal of the fixed appliance (Figure 10).

## 7. Discussion

Incorrect estimations of the center of resistance of #13 with the use of unmitigated forces led to the alveolar dehiscence on #13 in this case. Bracket inversion, use of individual tooth torque on the wire, and placement of Warren springs or Goodman springs are alternatives suggested in the literature to correct the root angulations and place displaced teeth back into the alveolar housing [11, 12]. Unfortunately, the lack of adequate leveling and rotation on #13 made it impossible for a rectangular wire to be inserted into the slot to use the abovementioned torquing auxiliaries.

Removal of all active unmitigated orthodontic forces from the affected tooth has been known to improve the detrimental periodontal conditions [13]. Hence, it was best decided to remove all active forces from #13 in order to allow the root to physiologically move into the alveolar housing.

Excessive buccal or labial movement of the teeth into the labial cortical plate may cause thinning of the gingiva and a partial see through of the underlying root contour giving it a so called “washboard effect” and resultant bone loss or root resorption as teeth contact the cortical bone [14].

Wainwright [15], Karring et al. [16], and Thilander et al. [17] demonstrated the occurrence of dehiscence or fenestrations of the buccal alveolar plate on labial movement of teeth and subsequent healing and reforms when the tooth moved back into the alveolar housing. Only Engelking and Zachrisson [18] demonstrated no recovery of the recessed gingiva after the occurrence of dehiscence or fenestration, following movement back into the alveolar housing.

Cases like these suggest that the etiology of crowded permanent teeth is simply too much tooth structure for the available arch length. Teeth are forced to erupt out of the arch form when inadequate space is available, but they will spontaneously drift into a good arch form when adequate space is provided [19].

Although there were many challenges during the course of this case, it was finished well in less than 2 years, and furthermore, a one-year follow-up shows stable results and no gingival pathology w.r.t. #13.

On retrospective analysis, one of our treatment alternatives, in which we had planned the extraction of #15, could have possibly been a better option. The idea of retracting #14 using a segmented T loop with high alpha activation (to keep the root of #14 away from that of #13) for retraction would probably make more sense as it would cause simultaneous traction of #13 as well, due to the “pull” effect of the transseptal fibers [20]. As there would be no direct force application on #13, the chances of unwarranted forces causing excessive tipping and buccal root movements would be nullified, in turn simplifying the treatment. Once #14 was completely retracted, #13 should have been in a much better occlusal position to be involved with a simple overlay wire for vertical traction. This plan was initially not finalized upon because of the considerations of time. It was thought that extracting #14 may be a better option as it was closer to the ectopic canine and would warrant quicker correction.

## 8. Conclusion

This case demonstrates observations of such physiologic movements of the root into the alveolar bone when space was created. Extracting or making space to let the teeth out of the arch spontaneously align, depends on the fact that self-correction or self-separation occurs so rapidly. Orthodontists should be aware of possible complications and solution strategies whenever mechanics do not deliver desired outcomes. It is also prudent that clinicians analyze their completed cases on the basis of learning objectives for the future, after assessing both efficiency and efficacy of strategies employed.

## Figures and Tables

**Figure 11:**
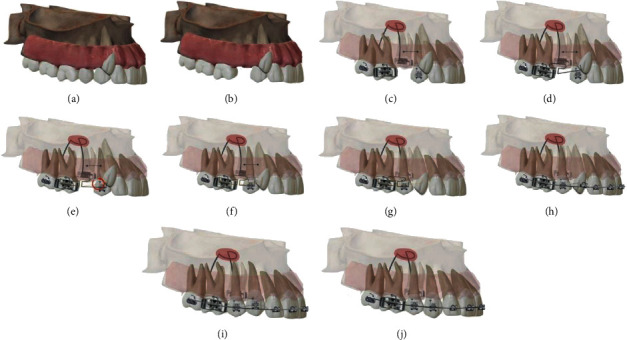
Illustration showing simulated movement of the ectopic canine into occlusion in a similar scenario following extraction of #15 (b). A segmented T loop being used for retraction of #14 into the extraction space and the transseptal fibers of #13 following #14 into occlusion (c–g). Overlay archwire to derotate and get #13 into alignment (h, i). Finishing on a 0.019^″^ × 0.025^″^ stainless steel archwire.

**Table 1:** Cephalometric analysis.

Values	Pretreatment	Posttreatment
Skeletal:		
SNA	78°	79°
SNB	70°	72°
ANB	8°	7°
SN-MP	41°	40°
FMA	34°	33°
ANS-GN	68 mm	66 mm
Dental:		
U1-NF	98°	105°
IMPA	97°	99°
Soft tissue:		
U lip-E line	-0.5 mm	1.2 mm
L lip-E line	1.4 mm	0.3 mm

## Data Availability

No archiving is available. All data is in the manuscript.
